# Long-term treatment with Perampanel of Chinese patients with focal-onset seizures, especially in sleep-related epilepsy: a prospective real-world observational study

**DOI:** 10.3389/fneur.2024.1364295

**Published:** 2024-02-29

**Authors:** Ye Xu, Qinyue Wang, Yufei Zhang, Yuncan Chen, Lan Xu, Guoxing Zhu, Chunlai Ma, Xunyi Wu

**Affiliations:** ^1^Department of Neurology, Huashan Hospital, Fudan University, Shanghai, China; ^2^Department of Pharmacy, Huashan Hospital, Fudan University, Shanghai, China; ^3^National Center for Neurological Disorders, Shanghai, China

**Keywords:** Perampanel, efficacy, safety, retention rate, sleep-related epilepsy, concentration

## Abstract

**Background:**

There is currently a lack of studies examining the long-term therapeutic effectiveness of the third-generation anti-sezure medication, perampanel (PER), for focal-onset seizures (FOS), particularly in Chinese patients with sleep-related epilepsy (SRE). Additionally, the appropriate dosage, plasma concentration, and the relationship between dose and plasma concentration of PER in Chinese patients are still uncertain.

**Methods:**

A prospective, single-center, 24-month observational study was conducted in patients diagnosed with FOS, with a focus on patients with SRE. Changes in seizure frequency from baseline, adverse events, and retention rates were analyzed at 12 and 24 months following the start of the treatment. Tolerability was evaluated based on adverse events and discontinuation profiles. PER plasma concentrations were used to assess dose-concentration-response relationships.

**Results:**

A total of 175 patients were included (median age: 25 years; range: 4–72 years; 53. 1% males and 46.9% females), with the SRE population accounting for 49. 1% (*n* = 86). The patients diagnosed with SRE showed considerably higher response rates than those who did not have this diagnosis (*p* = 0.025, odds ratio = 3.8). Additionally, the SRE group adhered better to PER treatment (*r* = 0.0009). Patients with a shorter duration of epilepsy (median: 3 years; range:2–7 years) demonstrated a more favorable therapeutic response to PER (*p* = 0.032). Throughout the administration of maintenance doses, among the entire FOS population, the concentration of PER (C0) ranged between 101.5 and 917.4 ng/mL (median, 232.0 ng/mL), and the mean plasma concentration of PER in the responders was 292.8 ng/mL. We revealed a linear relationship between PER dose and plasma concentration, regardless of whether PER was used as monotherapy or add-on therapy. The retention rates were 77.7% and 65. 1% at 12 and 24 months, respectively. Drug-related adverse events occurred in 45.0% of the patients and were mostly manageable.

**Conclusion:**

PER effectively reduced seizure frequency in Chinese patients with FOS, particularly in those with SRE, over a 24-month period. The treatment was well-tolerated and had a clear linear dose-plasma concentration relationship.

## 1 Introduction

Epilepsy is a widespread neurological condition, affecting individuals of different ages, races, social classes, and geographic locations. Focal-onset seizures (FOS) are the predominant manifestation of epilepsy and occur in ~60% of such patients (1). Early studies indicated that many focal seizures occur during sleep; such seizures are also focal onset in ~80% of cases (2). Moreover, seizures occurring during sleep increase the likelihood of another seizure event within the next 2 years (3), so the treatment of FOS, especially in sleep-related epilepsy (SRE), requires correct diagnosis and urgent medical intervention.

The third-generation anti-seizure medication (ASM) perampanel (PER) is a selective and non-competitive antagonist of the α-amino-3-hydroxy-5-methyl-4-isoxazolepropionic acid (AMPA) type of glutamate receptors (4). PER is rapidly and completely absorbed after oral administration, usually before bedtime, and only one daily dose is required (5). Phase III studies have shown the usefulness of PER as an adjunctive treatment, owing to its anti-seizure effects, in Asian patients (6) with FOS defined according to the 2017 International League Against Epilepsy (ILAE) classification (7).

To the best of our knowledge, treatment of SRE with PER and the relationship between PER plasma concentration and clinical efficacy in real-world practice have rarely been reported in studies of Chinese patients. Most of these studies were retrospective and had a relatively short observational period of 6–12 months (8–10). Simultaneously, the range of blood concentrations required for the effective treatment with PER has not been conclusively established, especially in Chinese patients.

To investigate the efficacy and safety of PER in Chinese patients with FOS, particularly those with SRE, as well as to explore the relationship between PER concentration and its effects, we designed a long-term, prospective real-world study. We believe that this study provides useful reference information for the clinical application of PER in China.

## 2 Methods

### 2.1 Study design

This was a prospective, single-center, long-term, observational study conducted in a real-world setting. Patients attending the outpatient or inpatient services of the Neurological Unit of the Huashan Hospital (Shanghai, China) between March 2021 and March 2023 were enrolled. The study protocol was approved by the Ethics Committee of the Huashan Hospital (KY2021-784) and followed the principles of the Declaration of Helsinki.

Patients at least 4 years of age or older diagnosed with FOS with or without focal to bilateral tonic-clonic seizures (FBTCS) were included. FOS was diagnosed according to the 2017 ILAE classification (7). Diagnosis of SRE was based on clinical and electroencephalographic findings (11). The enrolled patients regularly attended outpatient services. Patients or their legal representatives signed the informed consent form.

The exclusion criteria were as follows: (1) age ≤ 4 years; (2) severe progressive diseases of the central nervous system, severe circulatory system diseases, hematologic diseases, and immunocompromised conditions affecting follow-up; (3) paroxysmal non-epileptic seizures; (4) pregnancy or lactation; and (5) refusal to be included in this study.

For analysis purposes, patients were classified as having focal aware seizures (FAS), focal impaired awareness seizures (FIAS), or FBTCS, based on the 2017 ILAE classification (7). The sub-analysis was also performed to compare cases of SRE and non-SRE. Effects of the following clinical factors were compared: early add-on therapy with PER (≤2 concomitant ASMs at baseline) vs. late add-on therapy (≥3 concomitant ASMs) and therapy with or without enzyme-inducing ASMs (EIASMs). Concomitant ASMs were divided into EIASMs, including carbamazepine and oxcarbazepine, and non-EIASMs (any other ASMs). These drugs were included as EIASMs on the basis of their pharmacokinetic profiles.

During the study, patients received treatment with constant doses of approved ASMs and did not change the ASM types. We determined the steady-state PER concentration after the patient remained on the same PER dosing schedule for at least 21 days. The plasma concentration of PER was measured using high-performance liquid chromatography–tandem mass spectrometry method as reported previously (12).

### 2.2 Data collection

Data were collected from the patients’ medical records and seizure diaries. The following data were collected at baseline: demographics, age at epilepsy onset, epilepsy duration, etiology, number of concomitant ASMs, seizure types, as well as electroencephalographic and neuroradiological features.

Seizure frequency was calculated at baseline, at visits (V) V3, V6, V12, V18, and V24, as the mean number of seizures per month.

To reduce the fraction of those lost to follow-up, we also established telephone contact with patients to record their treatment status, prognosis, and adverse events (AEs).

### 2.3 Study variables

The primary efficacy endpoints were the proportion of patients who were seizure-free at 24 months, the median percentage of seizure reduction at 24 months, the proportion of responders (patients with ≥50% seizure reduction from baseline) at 24 months, and the retention rate on PER at 24 months.

The secondary efficacy endpoints included the proportions of patients who were seizure-free at 12 months, and the proportions of responders at 12 months.

During the studies, patients received ongoing treatment with stable doses of approved ASMs, and did not transform kinds of ASMs. We determined the steady-state concentration after the patient had remained on the same PER dosing schedule for at least 21 days.

Safety endpoints included the proportions of patients with drug-related AEs at 3, 6, 12, 18, and 24 months, if any; AE severity graded according to the Common Terminology Criteria for Adverse Events; and the proportion of patients with AEs that led to discontinuation of PER at each visit.

### 2.4 Statistical analysis

SPSS software (version 25.0; IBM, Armonk, NY) was used for statistical analysis. Continuous, normally distributed data are expressed as the mean ± standard deviation. Comparisons between two groups were performed using the *t*-test for independent samples. Non-normally distributed data are described as the median and quartile intervals, and comparisons of such data between two groups were performed using the Mann–Whitney test. Categorical data are expressed as frequencies and percentages (n, %), and the Pearson’s *χ*^2^ test or the Fisher’s exact test were used for subgroup analyses. The Wilcoxon test was used to analyze seizure frequency. All tests were two-sided, and differences were considered statistically significant at *p* < 0.05. Logistic regression was used for the multifactorial analysis of response rate differences. The discontinuation of PER for different subgroups was plotted according to the Kaplan–Meier method to estimate the retention rates and duration of PER use. The last-observation-carried-forward method was used for missing data (<5%).

## 3 Results

### 3.1 Patient distribution and disease characteristics

**Figure 1:**
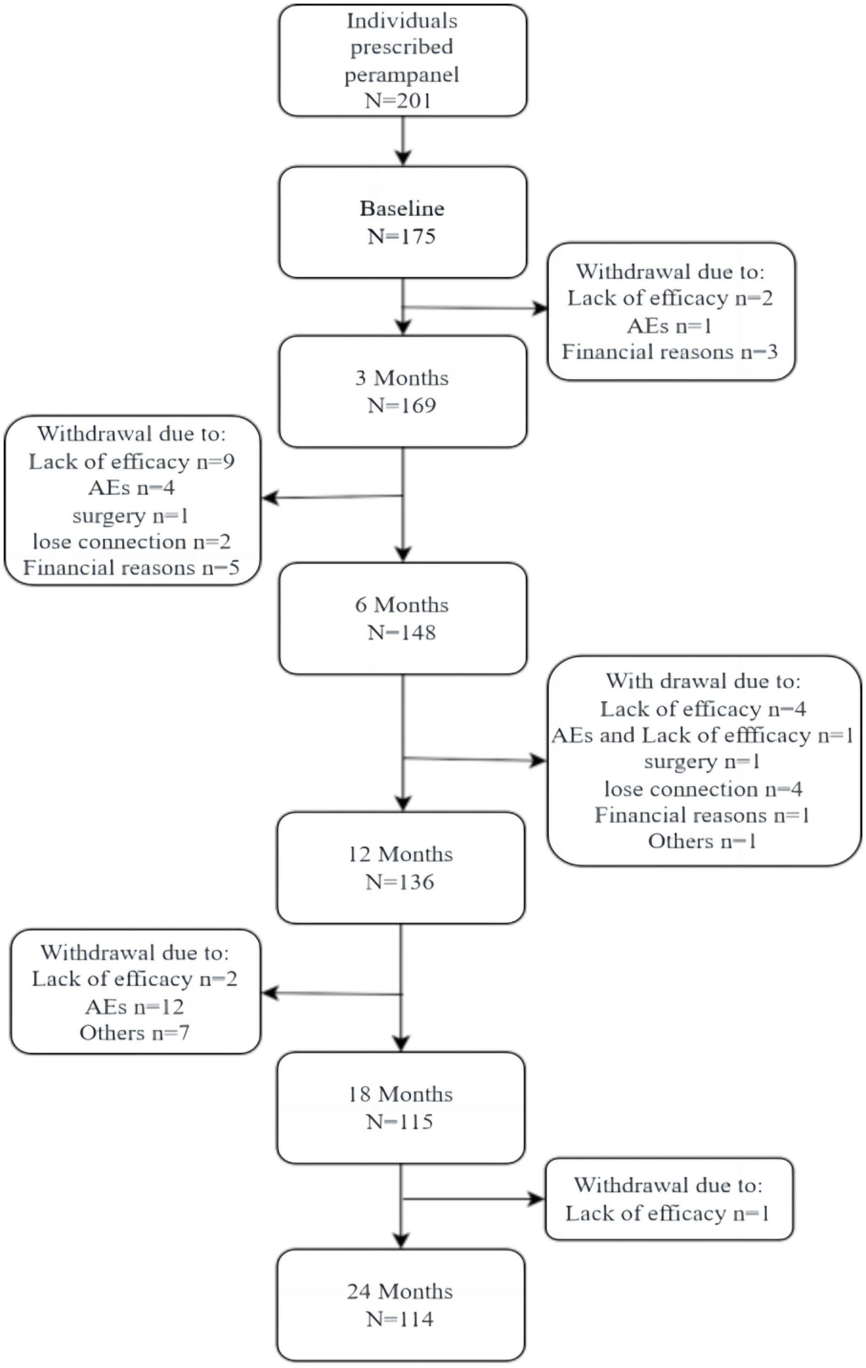
Patient flow chart from baseline to 24 months. AEs, adverse events.

A total of 175 patients were enrolled (Figure 1), including 93 males and 82 females, with a male-to- female ratio of 1. 14:1. The age range was 4–72 years (35.4% were 4–18 years old), median age was 25 years. Baseline characteristics are illustrated in Table 1.

**Table 1:** Patient baseline demographic and disease characteristics.

Index	Total *n* = 175
Sex, n (%)	
Male	93 (53. 1%)
Female	82 (46.9%)
Age, years	25 (18–35)
Onset age, years	16 (10.5–23)
Duration of epilepsy, years	5 (2–12.5)
Weight, kg	60 (54–72)
Height, m	1.65 (1.6–1.73)
BMI, kg/m^2^ (mean ± SD)	22.76 ± 0.27
Numbers of concomitant ASMs, n (%)	
0	34 (17. 1%)
1	62 (35.4%)
2	54 (30.9%)
3	21 (12.0%)
4	3 (1.7%)
5	3 (1.7%)
FAS patients, n (%)	23 (13.61%)
FAS/month	4 (1–30)
FIAS patients, n (%)	63 (37.3%)
FIAS/month	3 (1–7.56)
FBTCS patients, n (%)	123 (72.8%)
FBTCS/month	1 (0.48–3)
SRE, n (%)	86 (49. 1%)
Late add-on, n (%)	46 (26.3%)
Early add-on, n (%)	95 (54.3%)
Monotherapy, n (%)	34 (19.4%)
Concomitant EIASMs, n (%)	86 (49. 1%)
Concomitant sodium channel blockers, n (%)	96 (54.9%)
Concomitant LEV, n (%)	69 (39.4%)
Concomitant VPA, n (%)	40 (22.9%)
Abnormal EEG/VEEG, n (%)	85 (49. 1%)
Abnormal MRI, n (%)	27 (15.4%)
Etiology	
Structure	7 (4.0%)
Trauma	15 (8.6%)
Infection	17 (9.7%)
Tumor	39 (22.3%)
Vascular	23 (13. 1%)
Unknown	74 (42.3%)

ASM, anti-seizure medication; BMI, body mass index; EEG, electroencephalogram; EIASM, enzyme-inducing anti-seizure medication; FAS, focal aware seizures; FBTCS, focal to bilateral tonic–clonic seizures; FIAS, focal impaired awareness seizures; LEV, levetiracetam; MRI, magnetic resonance imaging; SD, standard deviation; SRE, sleep-related epilepsy; VEEG, video-electroencephalogram; VPA, valproic acid.

Overall, there were 175 and 114 patients in the follow-up set and completer cohort, respectively. The safety dataset included 175 patients.

### 3.2 Efficacy

In this study, 34 (19.4%), 95 (54.3%), and 46 (26.3%) patients were treated with PER as monotherapy, early add-on therapy, and late add-on therapy, respectively (Table 1). The response rates were 77.21 and 84.21% at 12 and 24 months, respectively.

Overall, the proportion of seizure-free patients increased from 48.53% at 12 months to 52.63% at 24 months.

At baseline, 3 months prior to the administration of PER, all patients presented with at least one type of FOS, and the diagnosis of SRE was based on clinical records before initiating PER. Table 1 illustrates the range of seizure frequencies observed at treatment initiation and the proportion of patients diagnosed with SRE.

At 24 months, no differences were observed in sex, mean age, body mass index, or number of seizure types between responders (*n* = 94) and non-responders (*n* = 20). The proportions of patients aged <18 years were also similar (Table 2).

**Table 2:** Responder and non-responder subgroup analysis at 24 months.

Index	Non-responders (*n* = 20)	Responders (*n* = 94)	*p*-value
Sex			
Male, n (%)	9 (50.0%)	56 (58.3%)	0.512
Female, n (%)	9 (50.0%)	40 (41.7%)	
Age (mean ± SD, years)	26 ± 13.43	27.48 ± 14.93	0.683
BMI (mean ± SD, kg/m^2^)	23.43 ± 2.89	22.98 ± 3.93	0.628
Onset age (median, range)	13 (9–15.75)	17 (12–26.25)	0.012*
Duration of epilepsy,(median, range)	7 (3–18.25)	3 (2–7)	0.032*
FAS, n (%)	3 (15.00%)	12 (12.77%)	0.240
FIAS, n (%)	7 (35.00%)	35 (37.23%)	0.063
FBTCS, n (%)	10 (50.00%)	68 (72.34%)	0.042*
Concomitant ASMs, n (%)			
VPA	5 (16. 13%)	25 (19.08%)	0.343
LEV	6 (19.36%)	43 (32.82%)	0.037*
OXC	6 (19.36%)	31 (23.66%)	0.088
CBZ	3 (9.68%)	15 (11.45%)	0.406
LTG	4 (12.90%)	2 (1.53%)	0.341
TPM	5 (16. 13%)	10 (7.63%)	0.352
LCM	2 (6.45%)	5 (3.82%)	0.256
PER therapy, n (%)			0.048*
Late add-on	9 (45.00%)	22 (23.40%)	
Early add-on	7 (35.00%)	60 (63.83%)	
Concomitant EIASMs, n (%)	8 (40.00%)	44 (46.81%)	0.579
Concomitant sodium channel blockers, n (%)	10 (50.00%)	47 (50.00%)	>0.999
Abnormal EEG/VEEG n (%)	13 (59. 1%)	72 (81.8%)	0.053
Abnormal MRI, n (%)	4 (20.00%)	18 (19. 15%)	0.734
Etiology			0.242
Structure	1 (3.57%)	2 (2.33%)	0.300
Trauma	4 (14.29%)	6 (6.98%)	0.300
Infection	5 (17.86%)	4 (4.65%)	0.932
Tumor	3 (10.71%)	22 (25.58%)	0.043*
Vascular	4 (14.29%)	12 (2.33%)	0.796
Unknown	11 (39.29%)	40 (46.51%)	0.578
Maintenance dose of PER (mean ± SD, mg/day)	3.45 ± 1.43	3.68 ± 1.58	0.511
Maintenance dose of PER (range, mg)	2–12	2–12	0.239
Age (years)			0.713
4–18	7 (35.00%)	32 (34.04%)	
>18	11 (65.00%)	62 (65.96%)	
Number of seizure types, n (%)			0.760
1	15 (75.00%)	75 (79.79%)	
≥ 2	3 (25.00%)	21 (22.34%)	

ASM, anti-seizure medication; BMI, body mass index; CBZ, carbamazepine; EIASM, enzyme- inducing anti-seizure medication; FAS, focal aware seizures; FBTCS, focal to bilateral tonic–clonic seizures; FIAS, focal impaired awareness seizures; LCM, lacosamide; LEV, levetiracetam; LTG, lamotrigine; OXC, oxcarbazepine; PER, perampanel; TPM, topiramate; VPA, valproic acid; *p < 0.05; The bold values means *p* < 0.1.

**Figure 2:**
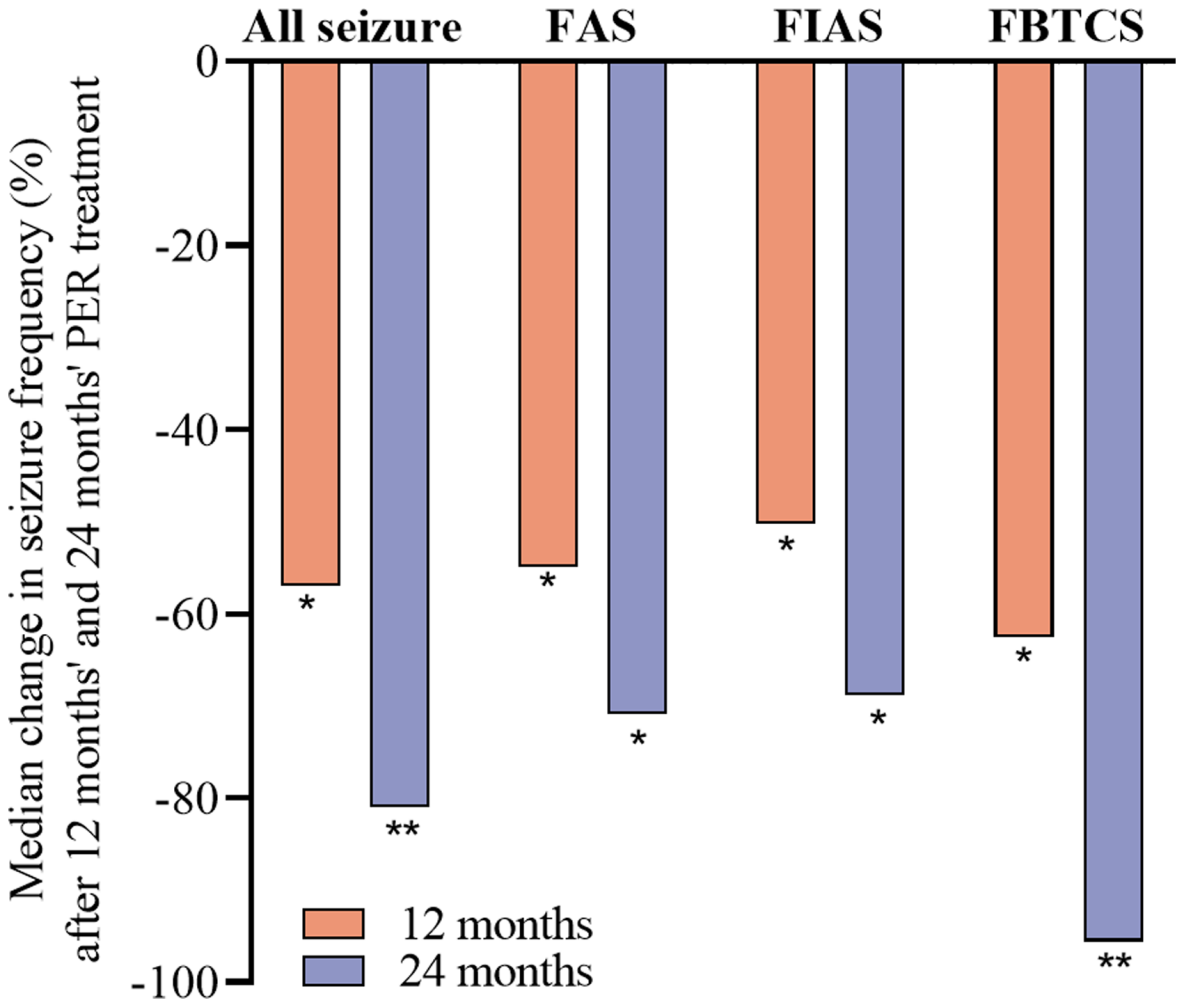
Median percentage of seizure reduction with perampanel at 12 and 24 months by seizure type. Statistical significance of differences from baseline is indicated as follows: *p < 0.01; **p < 0.001.

After 24 months, the median seizure frequency of each subtype was significantly reduced compared to that at baseline (*p* < 0.01 for FAS and FIAS; *p* < 0.001 for FBTCS). The percentage reductions were 81.0% for all seizures, 70.9% for FAS, 68.9% for FIAS, and 95.6% for FBTCS (Figure 2).

#### 3.2.1 Efficacy in the add-on group

At 12 months, the percentages of seizure-free patients were 50.0, 44.2, and 67.5% in the FAS, FIAS, and FBTCS groups respectively, and these proportions increased at 24 months. The fraction of seizure-free patients was higher in the FBTCS group than that in the FIAS group at 12 months (*p* = 0.021) and 24 months (*p* = 0.03).

At 24 months, the early add-on group (*n* = 60) had a higher response rate than the late add-on group (*n* = 22) (63.8% vs. 23.4%, respectively, *p* = 0.048, Table 2). Similarly, the early add-on group had a higher proportion of seizure-free patients than that in the late add-on group (*p* = 0.03).

#### 3.2.2 Efficacy in the monotherapy group

The response rates in the PER monotherapy group were as follows: 70.0 and 75.0% at 12 and 24 months, respectively. The proportions of patients achieving seizure freedom after PER monotherapy varied over time and were 65.0 and 62.5% at the 12- and 24-month follow-up visits, respectively. Furthermore, in the monotherapy population, 75 percentage of patients were diagnosed with SRE.

#### 3.2.3 Treatment outcomes in patients with SRE

The response rates were significantly higher in patients with SRE (*n* = 68) than in the non-SRE group (*n* = 56) at 12 months (85.3% vs. 69.6%, *p* = 0.049) and 24 months (96.6% vs. 79.5%, *p* = 0.018).

**Figure 3:**
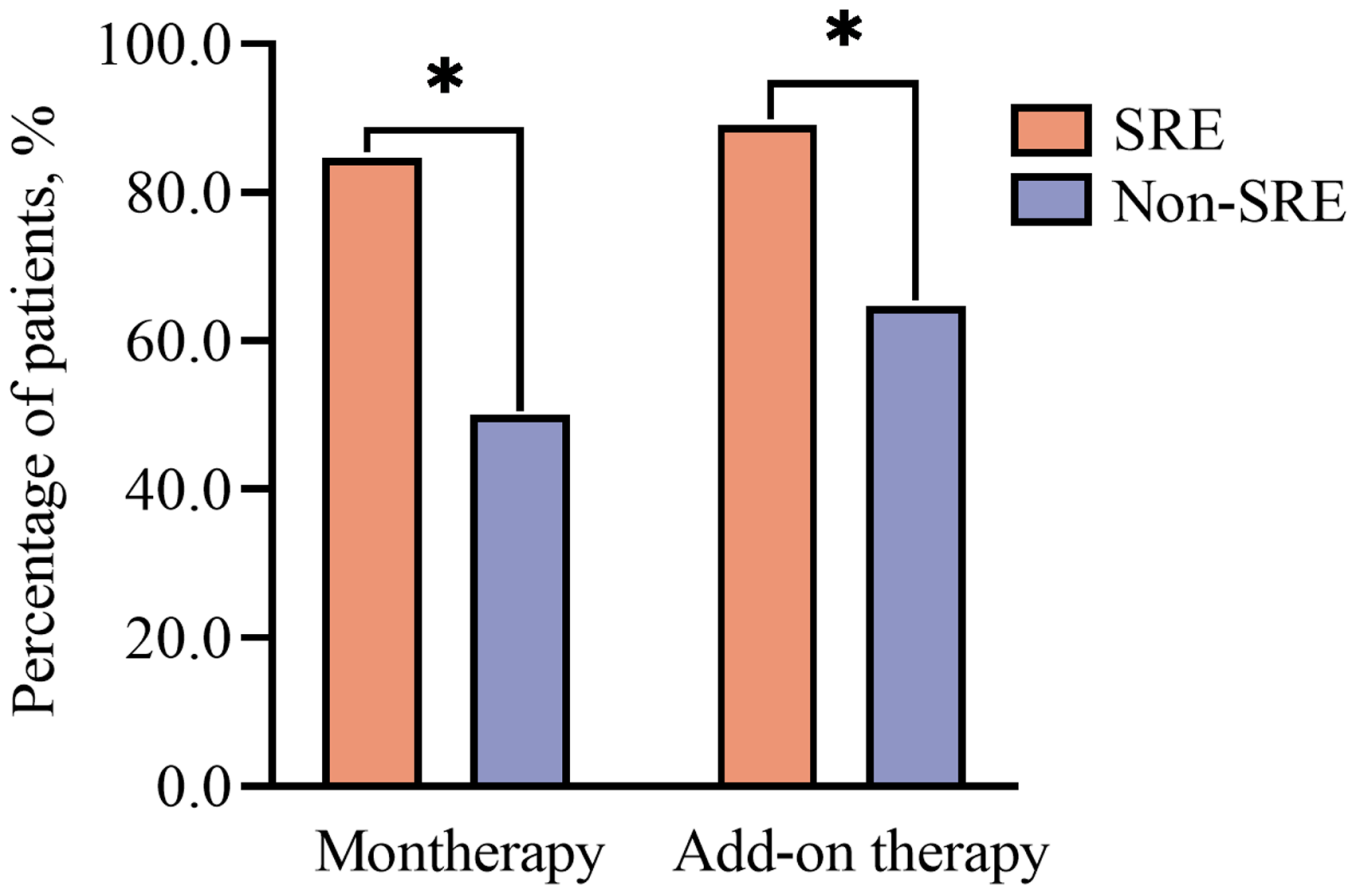
Response rate in patients with and without sleep-related epilepsy (SRE). *p < 0.05.

The fraction of patients that remained seizure-free at 24 months after PER monotherapy was consistently higher among individuals with SRE than among those with non-nocturnal epilepsy (84.6% vs. 50%, respectively, *p* = 0.04, Figure 3).

When PER was used as an add-on therapy, the fraction of patients achieving seizure freedom was also higher in the SRE group than in the non-SRE group at 12 months (62.7% vs. 39.3%, respectively, *p* = 0.049) and 24 months (89. 1% vs. 64.7%, respectively, *p* = 0.003, Figure 3).

#### 3.2.4 Outcomes of treatments with concomitant ASMs

The univariate analysis showed that patients who had three and more concomitant ASMs at baseline had lower response rates than those who had two or fewer concomitant ASMs (*p* = 0.006; Table 2).

Table 2 lists the common concomitant ASMs at 24 months, including a concomitant treatment with levetiracetam (LEV). In this study, when PER was used concomitantly with LEV, a high response rate was observed compared to that in patients who received other ASMs (*p* = 0.037; Table 2).

No differences in the rates of responses to sodium channel blockers (SCBs) were observed at 24 months. However, more PER-treated patients not receiving SCBs remained seizure-free compared to those receiving SCBs at 6 months (58.5% vs. 31.3%, respectively, *p* = 0.009) and 12 months (62.9% vs. 35.6%, respectively, *p* = 0.005).

At 24 months, the patients were categorized into two groups—those receiving EIASMs (49.7%) and those receiving other types of ASMs (50.3%). No significant differences were observed between these two groups in terms of the response rate (76.6% vs. 84.2%, *p* = 0.740) or fraction of patients that achieved seizure freedom (56.8% vs. 59.8%, *p* > 0.999). Similar results were obtained for the patients co-treated with valproic acid (VPA) and carbamazepine (Table 2).

#### 3.2.5 Logistic regression of patient-, disease-, and medication-related factors in responders and non-responders

**Figure 4:**
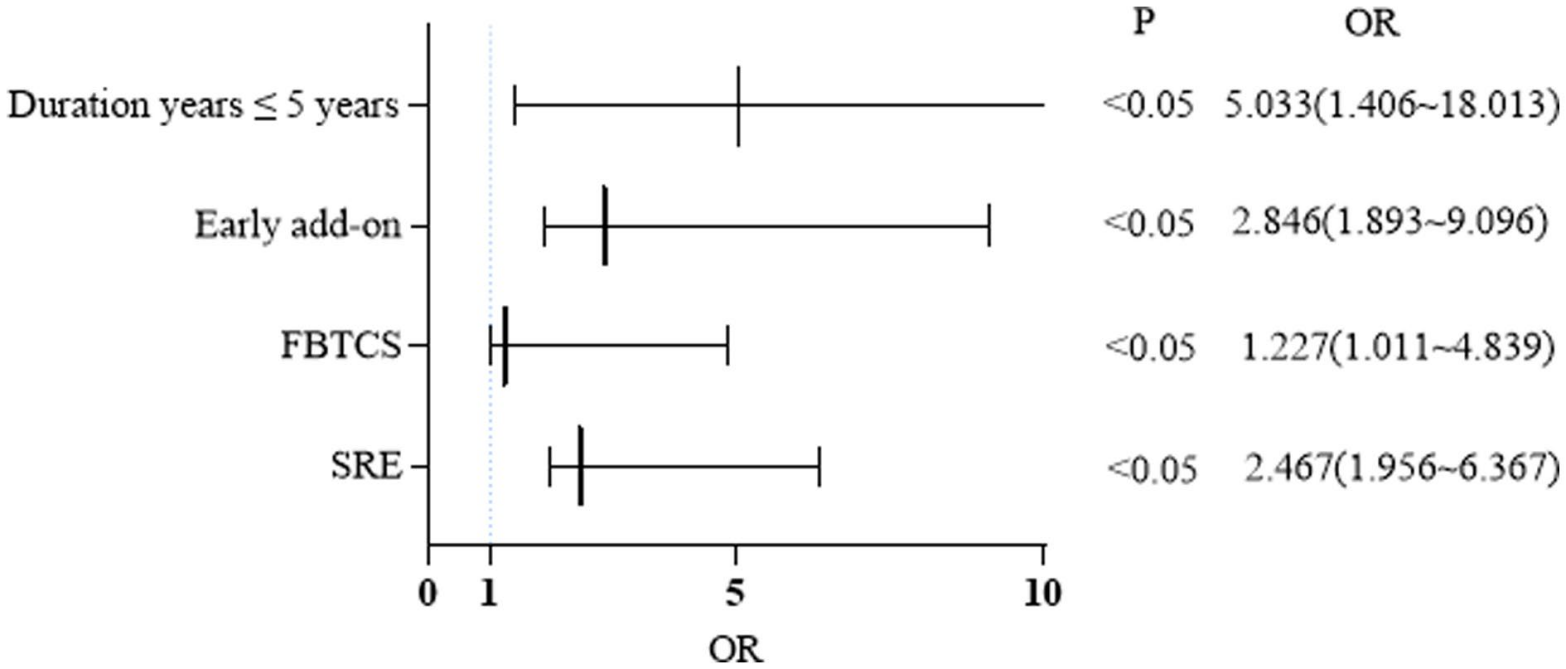
Binary logistic regression: factors influencing response rate at 24 months. FBTCS, focal bilateral tonic-clonic seizure; SRE, sleep-related epilepsy.

Table 2 displays the outcomes of all univariate analyses performed to identify baseline features that could differentiate responders (*n* = 94) from non-responders. Comparisons that met the set threshold (*p* < 0.1, as indicated in Table 2) were included in binary logistic regression to assess their predictive capacity for seizure control status. Figure 4 reveals that for patients with a short duration of disease and a diagnosis of FBTCS, especially those who experience nocturnal seizures, the addition of PER in the early stages of treatment has shown better efficacy.

### 3.3 PER dosage

The mean PER doses were 3.68 ± 1.58 and 3.45 ± 1.43 mg in the responder and non-responder groups at 24 months, respectively (*p* = 0.511).

The PER dose in the monotherapy group was 3.37 ± 1.48 mg (median: 4 mg) at the final checkpoint, and the most frequently administered dose in patients who achieved seizure freedom was equal or greater than 4 mg at the end of the follow-up.

**Figure 5:**
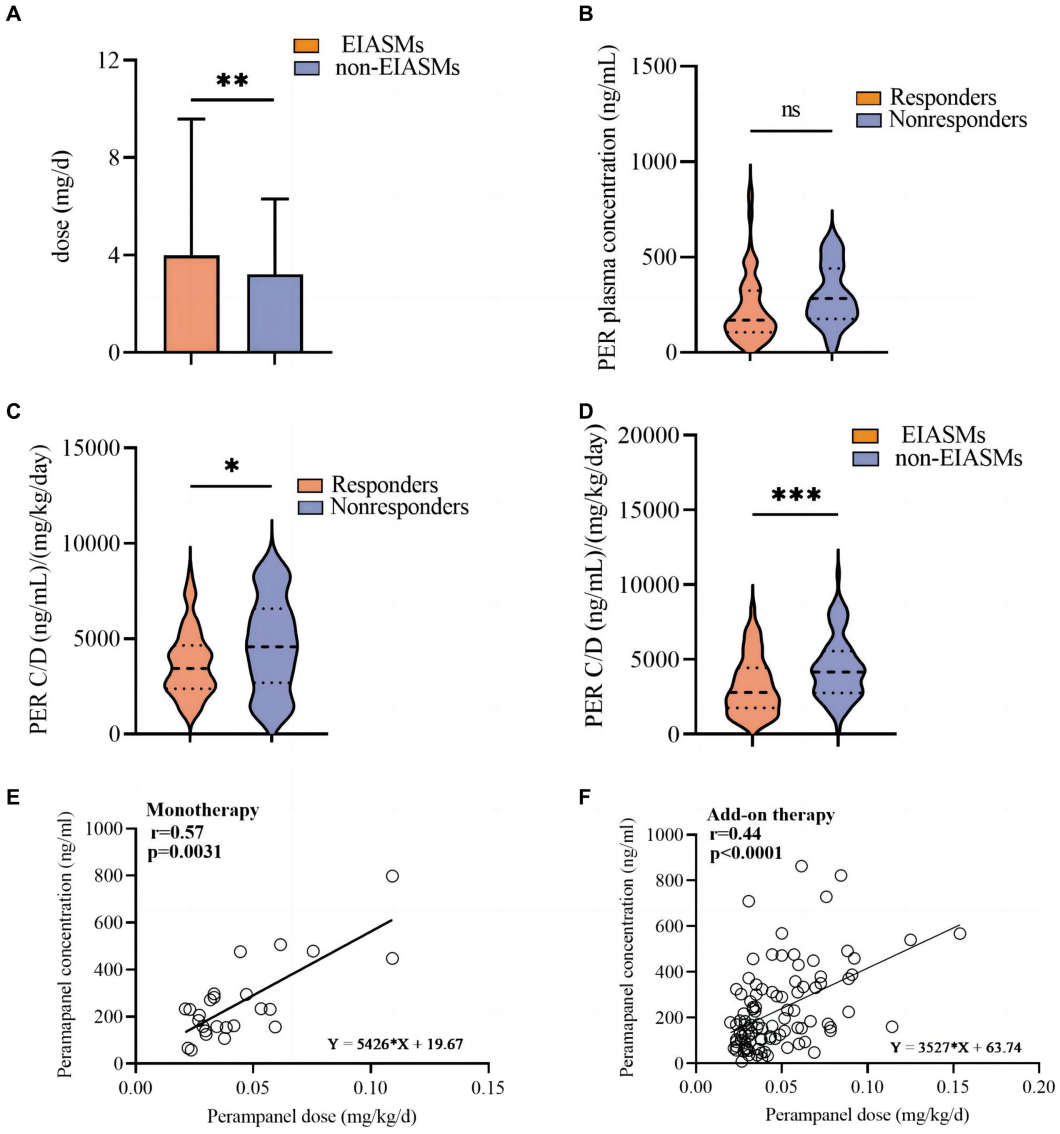
Analysis of perampanel dosage and plasma concentration among different subgroups. **(A)** Perampanel dose (mg/d) between EIASMs and non-EIASMs groups. **(B)** Perampanel plasma concentrations (ng/mL) in responder and non-responder groups. **(C)** Weight-adjusted perampanel plasma concentration (ng∙mL-1∙mg-1∙kg-1)in responder and non-responder groups. **(D)** Weight-adjusted perampanel plasma concentration (ng∙mL-1∙mg-1∙kg-1) in EIASMs and non-EIASMs groups. **(E)** The relationship between perampanel dose and weight-adjusted plasma concentration in the monotherapy population. **(F)** The relationship between perampanel dose and weight-adjusted plasma concentration in the add-on therapy population. Statistical significance of differences is indicated as follows: ns, no significance; *p < 0.05; **p < 0.01; ***p < 0.001.

Patients taking at least one EIASM when PER was started received a higher maximum PER dose than those who were not taking EIASMs (3.98 ± 5.60 mg vs. 3.21 ± 3.09 mg, *p* = 0.009, Figure 5A).

### 3.4 Plasma PER levels and efficacy

PER plasma concentration was measured in 114 patients with FOS (49 women, mean age, 34 ± 15 years, range, 10–69 years).

Among these patients, 94 (82.5%) were responders, with a mean PER plasma concentration of 292.8 ng/mL (median:249.6 ng/mL; range:107.4–917.4 ng/mL), which was numerically but not statistically higher (*p* = 0.42) than that in the non-responder group (median: 266.2 ng/mL, range: 47.82–596.4 ng/mL, Figure 5B). Weighted-adjusted PER concentration was significantly higher in non-responders compared with responders (4,686 ± 2,441 vs. 3,709 ± 1,746 ng·mL^− 1^·mg^− 1^·kg^− 1^, *p* = 0.012, Figure 5C).

The mean weight-adjusted plasma concentration of PER in patients concomitantly using EIASMs and non-EIASMs was 3,247 ± 1,930 and 4,857 ± 2,942 ng·mL^− 1^·mg^− 1^·kg^− 1^, respectively. Compared with non-EIASMs, EIASMs significantly reduced plasma PER concentration (*p* < 0.001, Figure 5D).

A linear relationship between PER dose and plasma concentration (*r* = 0.4908, *p* < 0.0001) was observed, and it remained significant after weight-adjusted dose was used (*r* = 0.4406, *p* < 0.0001). Furthermore, this correlation was also observed in the monotherapy group (*r* = 0.5671, *p* = 0.0031) and add-on therapy group (*r* = 0.4434, *p* < 0.0001) (Figures 5E,F).

### 3.5 Safety and influencing factors

During the 24-month follow-up period, AEs occurred in 42.8% of patients (*n* = 75). In 18 patients, AEs led patients to drop out from the study (Figure 1); 25.4% (*n* = 44) were mild, and 9.3% (*n* = 17) were moderate. The main AE was somnolence (29.0%). Dizziness occurred in 19.6% of patients, and 19.6% of patients had psychiatric symptoms, of which irritability accounted for half of the cases.

No severe psychiatric symptoms or deaths during treatment or 30 days after the last dose were noted in patients receiving 2–12 mg PER. One male patient with symptomatic epilepsy after head trauma treated with PER as secondary monotherapy experienced leg hair growth. He had been previously treated with VPA but did not take the drug for 2 years before treatment with PER.

#### 3.5.1 AEs and concomitantly used ASMs

The incidence of AEs did not significantly differ between patients who used EIASMs and those who did not (31.5% vs. 28.5%, respectively, *p* > 0.999). No significant differences were found in the incidence of AEs between patients who received SCB and those who received other ASMs (38.8% vs. 21.2%, *p* = 0.752) or between patients who did and did not receive VPA (33.5% vs. 26.5%, *p* = 0.482). Furthermore, no significant relationship was observed between AEs and the number of concomitant medications (*p* = 0.659).

There was no significant increase in psychiatric AEs in patients taking EIASMs compared to that in patients who were not taking EIASMs (51.9% vs. 48.8%, respectively, *p* = 0.584). A similar lack of elevated incidence of psychiatric AEs was noted in patients taking LEV (29.4% vs. 16.7% in patients who did not take LEV, *p* = 0.088).

#### 3.5.2 Dose and AEs

Patients who experienced AEs received significantly higher doses of PER than those who did not (4.08 ± 2.09 vs. 2.93 ± 1.92 mg, U = 2,751, *p* = 0.0077).

### 3.6 Retention rates and influencing factors

**Figure 6:**
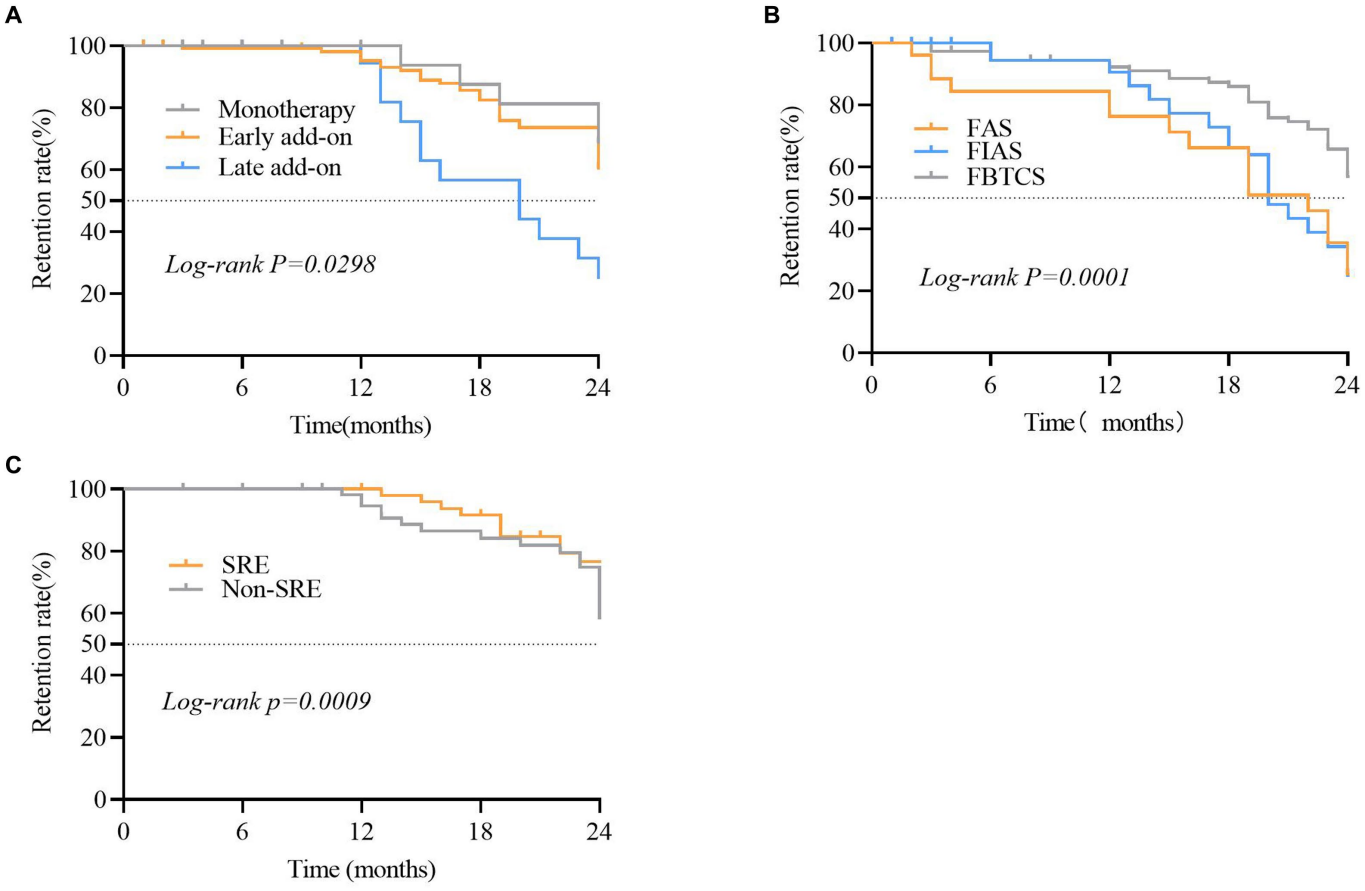
Comparison of the retention rate in different subgroups of patients that received perampanel (PER). **(A)** Focal aware seizures (FAS), focal to bilateral tonic-clonic seizures (FBTCS), and focal impaired awareness seizures (FIAS) subgroups. **(B)** Sleep-related epilepsy (SRE) and non-SRE subgroups. **(C)** Subgroups of patients that were treated with PER as monotherapy, early add-on, and late add-on. Statistical significance of differences is indicated as follows: *p < 0.05.

The PER retention rate was 77.7% (*n* = 136) and 65. 1% (*n* = 114) at 12 and 24 months, respectively. In patients that underwent monotherapy with PER, the retention rates were 60.0 and 46.6% at 12 and 24 months, respectively (Figure 1). The retention rates in the early add-on group were 80.0 and 60.5% at 12 and 24 months, respectively. In the late add-on group, the retention rates were 77.9 and 25.2% at 12 and 24 months, respectively (Figure 6). The primary reason for early withdrawal from PER treatment was the lack of efficacy.

The Kaplan–Meier retention analysis method was employed to plot discontinuation curves to compare PER treatment discontinuation. The early add-on group demonstrated favorable adherence to PER treatment compared to that in other patients (*p* = 0.044; Figure 6A). Significant differences were observed between patients belonging to various FOS subtypes, including FAS, FIAS, and FBTCS (*p* = 0.0001; Figure 6B). The retention rate among 72.8% of patients with FBTCS (*n* = 123) was significantly higher in the patients with SRE compared to that in the non-SRE group (76.7% vs. 56.5%, *p* = 0.0108; Figure 6C).

## 4 Discussion

We confirmed real-world PER safety and efficacy outcomes in a relatively large cohort of patients with FOS treated over a 2-year period. The efficacy rate in the present study was consistent with that in a recently published 3-year extension study (13). In that study, the 24-month responder and seizure-free rates were 84.4 and 56.3%, respectively, which are similar to the responder and seizure-free rates of 84.2 and 52.6% at 24 months achieved by the patients who completed the treatment in the present real-world study. Another study that analyzed the effects of PER as add-on therapy showed that nine of the 189 patients were seizure-free and in 33.4% of the patients, seizure frequency was reduced by ≥50% at 24 months (14). A possible explanation for the high proportion of patients who remained seizure-free in our study is that we used PER as an early add-on therapy in 54.3% of the patients. Additionally, patients that received PER as monotherapy or early add-on therapy had higher retention rates than those who received PER as late add-on therapy. Contrary to conclusions of a previous study (15), we found that 1-year retention rates were similar in patients that took one concomitant ASM and in those taking two or more concomitant ASMs.

The median percentage reduction in the frequency of seizures per 28 days in the FAS, FIAS, and FBTCS subgroups was above 50%. In the FBTCS subgroup, the median reduction was the highest: −62.5 and − 95.6% at 12 and 24 months, respectively, in line with the results of the OLEx study (16). PER antagonizes AMPA receptors and thus reduces the spread of epileptic discharge (17). Additionally, the FBTCS subgroup had a higher retention rate than the other two subgroups. This suggests that PER is particularly beneficial in patients with FBTCS.

Among the patients with FBTCS we found that the SRE group had a significantly higher response rate compared to that in the non-SRE group, suggesting that PER has a particularly favorable therapeutic effect in the patients with SRE. This finding is consistent with that of a previous clinical study conducted on patients with pharmacoresistant sleep-related hypermotor epilepsy (18). Additionally, the individuals diagnosed with SRE demonstrated higher adherence to PER treatment than those in the non-SRE group. Moreover, 75% of patients on monotherapy were diagnosed with SRE, and this subgroup had a higher rate of seizure freedom than the non-SRE group. This observation may be attributed to the timing of PER administration (before bedtime) as well as to the rapid and nearly complete absorption of PER after oral administration, low systemic clearance, and high relative bioavailability within the body (5). Previous studies have indicated that adequate control of seizures during sleep, particularly generalized tonic–clonic seizures, reduces the risk of sudden unexpected death in epilepsy (SUDEP) (2). This finding suggest that PER holds promise as an optimal ASM for managing SRE.

In a previously conducted clinical trial, PER exhibited a dose-dependent response, demonstrating significant effects at doses of up to 4 mg/day (19). Nonetheless, in our study, positive effects were seen at a lower mean dose of 3.68 mg/day. Similar data were reported in a study performed in.

Japanese patients (20). These results indicate that dosing needs to be individualized, and the individualized therapeutic range was important for ASMs including PER (21). However, the dose–concentration relationship and the reliable therapeutic range of PER in Chinese patients were largely unexplored. In our study, the mean overall plasma concentration was 295.3 ± 181.8 ng/mL, with a 75% range of 110.6–863.0 ng/mL. In phase III trials (22), plasma levels of PER in responders ranged between 180 and 980 ng/mL. In Chinese population, one study reported a 75% PER plasma concentration range of 180.0–610.0 ng/mL in children (23). Our study values were consistent with previously reported values, indicating that PER concentrations in our patients were within the presumptive therapeutic range. Additionally, a reduction in the frequency of seizures was observed in individuals who responded at a mean PER plasma concentration of 292.8 ng/mL, which was similar to the value of 266.2 ng/mL observed in non-responders, in line with previous studies (23, 24). The possible reason for this phenomenon is that most responders were administered PER early and received fewer previous/concomitant ASMs. Patients who were non-responders usually had more concomitant ASMs or started PER late in our hospital, most probably representing a refractory population.

Moreover, the weight-adjusted PER plasma concentration in non-responders was significantly higher than that in responders. Therefore, we suggest that individualized adjustment of PER dosage should be based on body weight and concomitant ASMs.

Our results support the findings of previous studies (25, 26), indicating that the efficacy and safety of PER remain consistent, regardless of the baseline use of EIASMs. However, patients who are prescribed EIASMs usually need a higher dose of PER than those who are not prescribed EIASMs, to achieve an effective treatment outcome. Additionally, the significant reduction in PER plasma concentration by EIASMs highlights the need for careful consideration of the dose-efficacy relationship and appropriate dosage adjustment when administering PER concomitantly with EIASMs.

We also examined the influence of other patient- or medication-related factors that could improve clinical outcomes or lower incidence of adverse events. Few studies have investigated this matter, although the FYDATA study reported better clinical responses to PER in patients aged ≥65 years with vascular diseases (9). In the present study, there were no significant differences observed between young and elderly patients in terms of achieving seizure control. However, we observed that patients with self-limited epilepsy with centrotemporal spike and predominantly nocturnal seizures showed favorable therapeutic efficacy with PER in the minor group, with all of them (*n* = 12) being responders and 75% being seizure-free at 24 months, which was consistent with a previous study (27).The implementation of binary logistic regression revealed that patients with a shorter epilepsy duration (median: 3 years, range: 2–7 years), limited prior exposure to other ASMs, and a diagnosis of FBTCS, particularly within the SRE group, displayed a significantly higher clinical response to PER.

Similar to second-generation ASMs, PER appears effective and safe for patients with brain tumor- related epilepsy. PER treatment allowed for a high rate of seizure control in these patients in the current and previous studies (28). Both current and recent real-world studies documented particularly effective seizure control in patients treated with both LEV and PER (29). Despite the concern about an increased risk of psychiatric AEs in patients treated with such a combination, neither our nor a previous study reported any corresponding evidence (30). Although more homogeneous and larger cohorts are needed to confirm these outcomes in patients with brain tumor-related epilepsy, it would also be interesting to evaluate the effect of PER on tumor progression (31). According to our findings, PER with LEV is an effective ASM combination in this patient group.

The overall PER safety profile in our study was similar to that in previous reports (32). Vellus hair growth on the legs occurred in one patient taking PER and VPA for treatment, which has not been Reported previously. PER-induced hair curling had been reported in a patient with epilepsy associated with Pitt–Hopkins syndrome treated with LEV and VPA (33). There is no obvious reason for this adverse event, but it is important to consider in patients treated with VPA when adding PER to their treatment regimens.

Our study has several strengths. First, it was a prospective and comprehensive analysis of the factors influencing the efficacy and retention of novel ASMs, including PER, in patients with FOS and we.

specifically focused on the SRE group. Second, PER is a promising medication for reducing the incidence of SUDEP. Third, patients were retained over the course of 24 months, and children with epilepsy syndromes were of concern, providing a reference for the long-term use of PER in a broader application of patients in Chinese clinical practice. Finally, we confirmed the linear relationship between PER dose and plasma concentration.

This was a real-world study; thus, it has all the limitations intrinsic to this type of observations, such as lack of randomization, ethical considerations, and et al. To confirm the therapeutic range of PER larger sample size and longer follow-up will be necessary in the future.

## 5 Conclusion

In summary, our study demonstrated the efficacy of PER in reducing seizure frequency in patients with FOS. The responder rate among the patients with FBTCS was higher in patients with SRE compared to that in the non-SRE group. We also observed a higher PER retention rate in the FBTCS group, particularly within the SRE subgroup. These observations suggest that PER could reduce the incidence of SUDEP. The retention rate for monotherapy or adjunctive therapy with PER in the Chinese population was comparable to that in Western countries. LEV was the most efficacious concomitant ASM, and its combination with PER did not increase AE incidence. The evaluation of factors associated with patients, diseases, or medication demonstrated that individuals with a shorter duration of epilepsy and those who had fewer previous ASMs, as well as those diagnosed with FBTCS, particularly within the SRE group, had a superior clinical response to PER. Prolonged use of PER did not increase the overall AE incidence. Further long-term follow-up studies are necessary to confirm the efficacy and tolerability of PER in patients with epilepsy.

## Data availability statement

The original contributions presented in the study are included in the article/Supplementary material, further inquiries can be directed to the corresponding author.

## Ethics statement

The studies involving humans were approved by the Ethics Committee of the Huashan Hospital. The studies were conducted in accordance with the local legislation and institutional requirements. Written informed consent for participation in this study was provided by the participants’ legal guardians/next of kin.

## Author contributions

YX: Conceptualization, Data curation, Formal analysis, Investigation, Methodology, Software, Supervision, Validation, Visualization, Writing – original draft, Writing – review & editing. QW: Data curation, Investigation, Writing – review & editing. YZ: Conceptualization, Data curation, Investigation, Methodology, Resources, Supervision, Writing – review & editing. YC: Data curation, Investigation, Writing – review & editing. LX: Conceptualization, Resources, Writing – review & editing. GZ: Data curation, Resources, Writing – review & editing. CM: Data curation, Funding acquisition, Investigation, Project administration, Resources, Supervision, Writing – review & editing. XW: Conceptualization, Data curation, Funding acquisition, Methodology, Project administration, Supervision, Writing – review & editing.

## Supplementary material

SUPPLEMENTARY FIGURE 1 Overall response of focal-onset seizures.

SUPPLEMENTARY FIGURE 2 Perampanel adverse reaction monitoring.
