# Supplementary figures and images for: Long-term treatment with Perampanel of Chinese patients with focal-onset seizures, especially in sleep-related epilepsy: a prospective real-world observational study
Source: Front Neurol. 2024 Feb 29;15:1364295. doi: 10.3389/fneur.2024.1364295 (PMC10937527; doi:10.3389/fneur.2024.1364295)

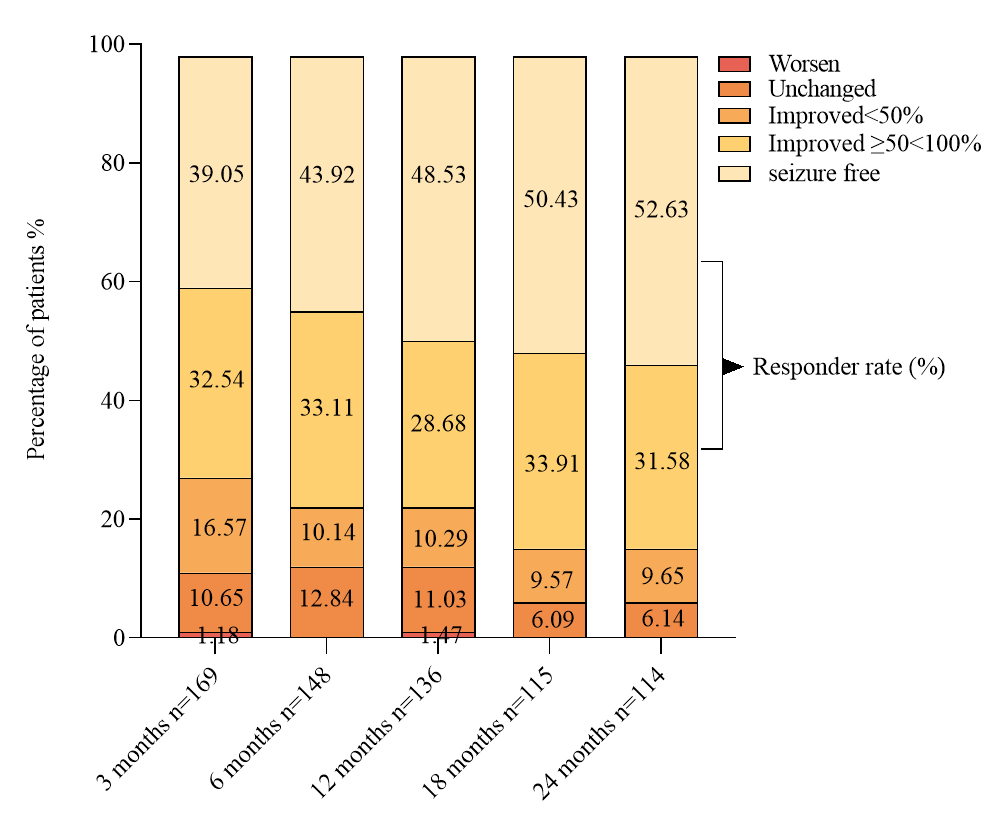

Supplement: SUPPLEMENTARY FIGURE 1 — Overall response of focal-onset seizures. [file Image_1.TIF]

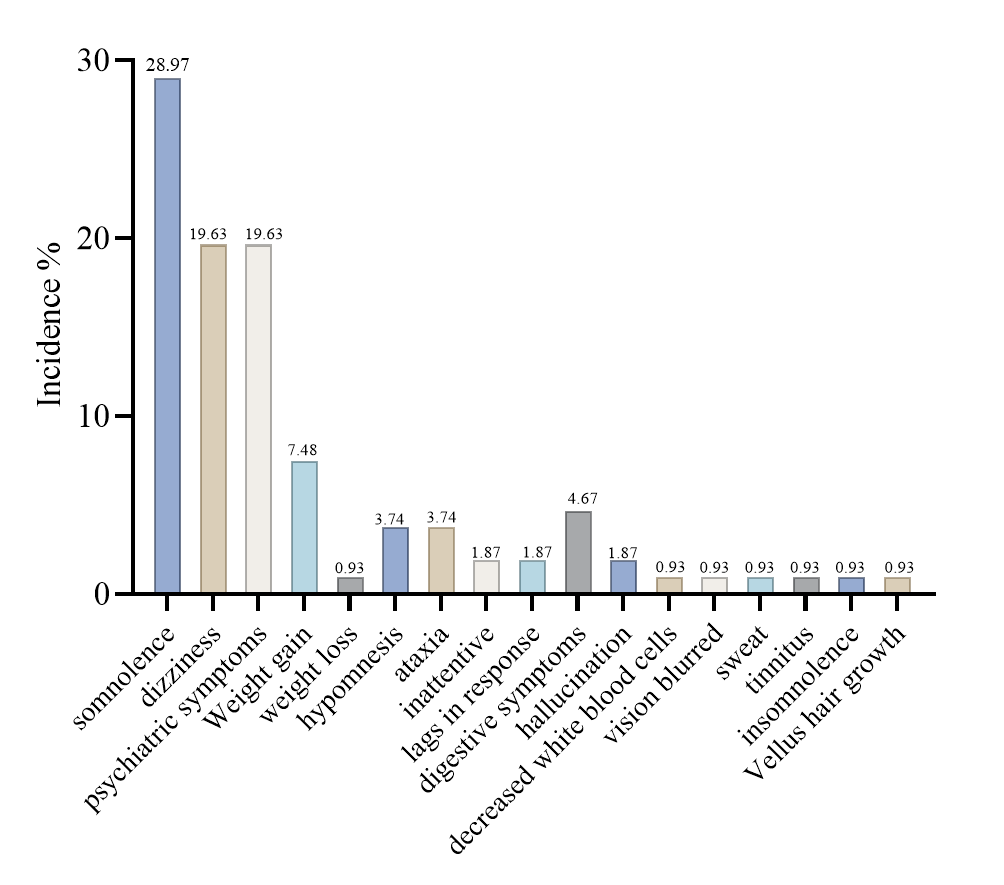

Supplement: SUPPLEMENTARY FIGURE 2 — Perampanel adverse reaction monitoring. [file Image_2.TIF]
